# Nurse-Coordinated Blood Pressure Telemonitoring for Urban Hypertensive Patients: A Systematic Review and Meta-Analysis

**DOI:** 10.3390/ijerph18136892

**Published:** 2021-06-27

**Authors:** Woo-Seok Choi, Nam-Suk Kim, Ah-Young Kim, Hyung-Soo Woo

**Affiliations:** 1 Moon Soul Graduate School of Future Strategy, Korea Advanced Institute of Science and Technology (KAIST), Daejeon 34141, Korea; journalist@kaist.ac.kr (A.-Y.K.); h.woo@kaist.ac.kr (H.-S.W.); 2 Keyu Internal Medicine Clinic, Daejeon 35250, Korea; 3 Public Health and Welfare Bureau, Daejeon City Hall, Daejeon 35242, Korea; heewasu@korea.kr

**Keywords:** blood pressure, hypertension, COVID-19, nurse, coordination, telemonitoring, urban

## Abstract

Coronavirus disease 2019 (COVID-19) has put hypertensive patients in densely populated cities at increased risk. Nurse-coordinated home blood pressure telemonitoring (NC-HBPT) may help address this. We screened studies published in English on three databases, from their inception to 30 November 2020. The effects of NC-HBPT were compared with in-person treatment. Outcomes included changes in blood pressure (BP) following the intervention and rate of BP target achievements before and during COVID-19. Of the 1916 articles identified, 27 comparisons were included in this review. In the intervention group, reductions of 5.731 mmHg (95% confidence interval: 4.120–7.341; *p* < 0.001) in systolic blood pressure (SBP) and 2.342 mmHg (1.482–3.202; *p* < 0.001) in diastolic blood pressure (DBP) were identified. The rate of target BP achievement was significant in the intervention group (risk ratio, RR = 1.261, 1.154–1.378; *p* < 0.001). The effects of intervention over time showed an SBP reduction of 3.000 mmHg (−5.999–11.999) before 2000 and 8.755 mmHg (5.177–12.334) in 2020. DBP reduced by 2.000 mmHg (−2.724–6.724) before 2000 and by 3.529 mmHg (1.221–5.838) in 2020. Analysis of the target BP ratio before 2010 (RR = 1.101, 1.013–1.198) and in 2020 (RR = 1.906, 1.462–2.487) suggested improved BP control during the pandemic. NC-HBPT more significantly improves office blood pressure than UC among urban hypertensive patients.

## 1. Introduction

Essential hypertension is the primary modifiable risk factor for cardiovascular disease, which is a leading cause of death according to the World Health Organization (WHO) [1]. Approximately 45% of American adults had elevated blood pressure in the 2017–2018 time period [2,3], but only 34% were being managed to bring the blood pressure down to the recommended treatment level [4]. It has been reported that managing blood pressure to remain within the recommended level reduced the incidence of stroke by 35–40%, heart attack by 20–25%, and heart failure by more than 50% [5]. Moreover, the American Heart Association/American College of Cardiology in 2017 and the European Society of Hypertension/European Society of Cardiology in 2018 issued guidelines recommending that the target blood pressure in hypertensive patients should be regulated more strictly than recommended by previous guidelines [6,7]. Additionally, recent studies have reported that essential hypertension is one of the most common comorbidities that cause lung damage and mortality due to the coronavirus disease 2019 (COVID-19) [8,9]. Thus, strict blood pressure management is crucial to reduce the incidence of cardiovascular disease (CVD) in patients with risk factors.

Despite preventive measures such as hand washing, self-isolation, mask wearing, and social distancing, COVID-19, which first emerged in Wuhan, China, spread rapidly in high-income countries such as the United States, Spain, Germany, Italy, and Korea in the early days of the pandemic [10]. Although the number of newly confirmed patients has been decreasing with the administration of the vaccine since December 2020 [11], the COVID-19 pandemic is still ongoing. According to the WHO, as of April 2021, there were approximately 1.6 billion cases of severe acute respiratory syndrome coronavirus 2 (SARS-CoV-2)—the virus that causes COVID-19—infections worldwide, including 3 million deaths [12]. However, with the exception of some countries, the global vaccination rate is still too low (below 40%) [12]. In particular, in most countries in Africa, the Middle East, and Southeast Asia, as of the end of April 2021, only approximately 10% of the population had received at least one dose of a vaccine [12]. The drastic increase in demand for medical care during the pandemic has exposed the limitations of traditional medical systems and is adversely affecting the existing medical systems for non-communicable diseases (NCDs) such as essential hypertension [13].

During infectious disease pandemics, a high frequency of direct contact between individuals in densely populated cities can increase the infection risk even in well-established in-person medical infrastructures [14]. Thus, the need for treatment through non-face-to-face interaction between doctors and patients is being emphasized more than ever before in cities [15]. Home blood pressure telemonitoring (HBPT) is widely used as an alternative measure in the management of hypertensive patients and is known to be effective in overcoming clinical inertia that may occur in face-to-face care settings [3,16,17,18]. In addition, remote monitoring of home blood pressure is helpful in managing patients by finding white-coat effects and masked hypertension that may be overlooked by doctors in medical institutions [19,20]. Studies on the effectiveness of nurse-coordinated HBPT (NC-HBPT) to prevent CVD attacks in hypertensive patients existed prior to the COVID-19 pandemic [21,22,23,24,25]. However, evidence on whether or not HBPT statistically enhances blood pressure control by improving communication between patients and healthcare providers has been mixed. Additionally, there is a paucity of literature indicating a solid basis for the effectiveness of nurse-led remote monitoring in the avoidance of CVD.

A coordinator plays a vital role in remote monitoring in chronic diseases [21,26], and nurses are core coordinators in the telemonitoring teams of medical institutions, working either independently or as members of a team [27]. Nurses can provide more regular follow-up, high-quality care, favorable health outcomes, and higher patient satisfaction, all equivalent to that achieved by physicians [28]. A 2005 review recommended nurse-led monitoring as an additional measure to support face-to-face therapy [29]. The literature classifies ancillary interventions to help control blood pressure into one of six categories: care led by health professionals, patient monitoring, education of medical professionals, education of patients, organizational interventions, and appointment reminder systems. Particularly, for the step-by-step care of hypertension that is not controlled by drug treatment, nurse-led regular monitoring was emphasized. However, there is uncertainty about the effectiveness of nurse-led remote monitoring in cities with established medical infrastructure and abundant medical resources. Although many studies have reported the nurses’ role in HBPT intervention [3,20,28,29,30,31], no study has systematically reviewed the effectiveness of NC-HBPT in urban areas and, particularly, none have presented quantitative results according to the temporal progress. Therefore, comprehensive comparative analysis of outcome data of NC-HBPT performed in urban areas before and during the COVID-19 pandemic is important to overcome the challenges of NCD and communicable disease (CD) management and to determine the future directions for remote monitoring policies.

This study hypothesizes that NC-HBPT in urban adult hypertensive patients is not more effective than usual care (UC) in preventing CVD and that its effect over time would be identical to that of UC. To derive robust results, we conducted a systematic review and meta-analysis of randomized controlled trials (RCTs), which are placed at the top in the hierarchy of evidence-based research. Previous meta-analyses investigated the effect of HBPT in hypertensive patients [32,33], but none have integrated the results of NC-HBPT for efficient implementation in cities yet. Thus, this study aimed to examine and compare the following: mean changes in systolic blood pressure (SBP) and diastolic blood pressure (DBP); rates of achieving the target blood pressure after NC-HBPT; and effects of NC-HBPT over time in an urban area.

## 2. Materials and Methods

### 2.1. Literature Search and Identification of Eligible Studies

We conducted a review in accordance with the guidelines summarized in the Cochrane Handbook for Systematic Reviews of Interventions [34]. A protocol for this study has been published in the PROSPERO [CRD42020222789], which is an international prospective register of systematic reviews operated by the Center for Reviews and Dissemination at the University of York [35]. Studies evaluating HBPT that were published between the date of inception of the utilized databases and 30 November 2020, were identified. The electronic databases we used included PubMed, EBSCOhost, and the Cochrane library (CENTRAL), and the search was limited to studies published in peer-reviewed journals in English. The related search keywords included “urban”, “hypertension”, “remote monitoring”, “telemonitoring”, “telemedicine”, and “randomized controlled trials.” The adopted search formula was constructed by combining free terms of relevant keywords and Medical Subject Headings (MeSH) terms via truncation, Boolean operators, and phrasing. We first searched in PubMed using this structured formula and sequentially performed additional searches according to the syntax of each database (Appendix A).

All retrieved data were exported to EndNote X8.2 (Thomson Reuters, Philadelphia, PA, USA). Titles and abstracts of each study were screened, and the main text was reviewed as required. We searched for all meta-analyses conducted previously on the topic and reviewed all primary studies and relevant references in those meta-analyses. To find grey literature, we referred to related websites in the United States and Europe (e.g., OpenGrey: http://opengrey.eu/ (accessed on 30 November 2020); Grey Literature Report: https://www.greylit.org/ (accessed on 10 December 2020). To ensure objectivity and transparency of the eligibility assessment, two of the authors (WSC and AYK) independently conducted the literature search and arrived at a mutually-agreed selection of studies.

### 2.2. Inclusion and Exclusion Criteria

An intervention group was defined as one in which patients measured their blood pressure on their own at home, reported the measurements to their doctors, and regularly visited the medical institute for follow-up, and in which a coordinator—including a nurse with or without other health care professionals—was involved in the process. As for nurses, only situations where the registered nurse monitored the patient’s home blood pressure using an automatic sphygmomanometer without face-to-face contact were included. All the following actions were also included: consultation with the patient using a telephone line, mobile phone, computer, or letter; education on the disease or intervention process; execution of accompanying interventions such as a text message reminder service; and sending information regarding the patient’s health status.

A control group was defined as one in which patients received routine in-person examinations at the doctor’s office. Since NC-HBPT is not yet a standardized treatment itself, none of the included studies provided an equivalent of NC-HBPT, and so no active control groups were included in our analyses. Their ethnicity, level of income, and severity of hypertension were not considered separately.

We included studies (1) involving patients with essential hypertension (SBP ≥ 130 mmHg, DBP ≥ 80 mmHg) regardless of hypertension onset or history of pharmacotherapy; (2) including patients who received treatment at an urban medical institution; (3) having an intervention that was provided for ≥2 months; (4) utilizing a RCT design; and (5) involving adults aged ≥17 years. The diagnostic criteria for hypertension in Europe are SBP ≥ 140 or DBP ≥ 90, which is higher than that in the US [7]. Thus, the diagnostic guideline for hypertension published by the 2018 ACC/AHA associations [6], which has a wider diagnostic range, was adopted in searching databases.

However, we excluded studies (1) conducted in several regions with unclear study locations; (2) having a research location that was a mix of urban and rural areas; (3) involving patients with acute CVD or stroke with a drastic change in blood pressure; (4) involving women in the peripartum period; (5) having an intervention provided to patients in nursing homes or care facilities; (6) having an intervention provided as part of another intervention program for a different disease; (7) using a different intervention in the UC group; and (8) utilizing a cluster- or cross-over RCT design. In this study, an “urban area” was defined according to the administrative functions and population size.

### 2.3. Study Selection

A further search was conducted by reviewing the full-text manuscripts of studies identified during the first round of screening and their reference lists. All articles related to “coordinator” and “nurse” were additionally identified. Among the blinded RCTs that regularly verified the effects of these two groups, those that reported changes in blood pressure measurements before and after interventions were selected for data synthesis.

Two of the authors (NSK and HW) independently classified and excluded duplicate studies and those that did not meet the inclusion criteria. The main body of potentially valid studies was reviewed, and any disagreement between the two authors was resolved by a senior author (WSC).

### 2.4. Outcomes

The primary outcome was the weighted mean difference (WMD) of office SBP and DBP between the baseline and follow-up in the NC-HBPT and UC groups. The secondary outcome was the rate of target blood pressure achievement.

### 2.5. Data Extraction and Coding

Two researchers (NSK and AYK) independently extracted data from the selected primary studies. The data were coded in an electronic sheet using Comprehensive Meta-Analysis Software Version 2.2.064 (CMA, Biostat, Englewood, NJ, USA). Patients’ age and sex, duration of remote monitoring (months), accompanying interventions, intervention pathway, the coordinator’s profession, and outcome data were extracted. When a single article had different follow-up periods for the intervention or different sample sizes, or compared two or more interventions [21,22,26,27,36,37,38,39,40], the results of each intervention were classified as independent comparative studies. For missing or inaccurate data in the primary research materials [27], we referred to journal websites or public trial registries (e.g., the US National Institutes of Health ongoing trials register) or directly contacted the authors. In cases where the standard deviation (SD) values for the mean changes were not presented [38], the values were imputed by calculating the mean of individual studies included in the review, and a sensitivity test was conducted to check for bias [40,41]. For the one study that did not provide baseline data [36], the data from the first assessment and the last follow-up were compared. In case of any disagreement between the researchers, a third researcher (WSC) adjudicated.

### 2.6. Quality Assessment

Two researchers (NSK and HW) independently assessed the risk of bias using Review Manager (RevMan, Version 5.4, Copenhagen: The Nordic Cochrane Center) by the Cochrane Collaboration [42], evaluating the RCTs in terms of selection, performance, detection, attrition, reporting biases, and other bias domains. A senior researcher (WCS) resolved any disagreement between the two researchers.

### 2.7. Quantitative Data Synthesis

Two researchers (WSC and NSK) analyzed the coded data. A random effects model (REM) was adopted because most of the primary studies were performed in different research institutions or by different researchers.

For computational options for data synthesis, WMD was set as a continuous variable and relative risk (RR) as a dichotomous variable; the changes in the mean office SBP and DBP of the NC-HBPT and UC groups before and after the intervention were extracted first. Hedges’ g (g) was additionally calculated to determine the appropriateness of the effect size. If the *g*-value converted from the WMD was at least 0.5, the effect size was deemed appropriate for analysis [43]. RR was used to determine the rate of achieving the target blood pressure and was calculated using the number of samples that reached the target blood pressure during each follow-up period. All results are reported with 95% confidence intervals (CIs), and CMA software was used for statistical analysis.

Overall statistics, including weighted values, were analyzed by combining independent data. A χ^2^ test was used to assess differences between studies, and a *p*-value < 0.10 was deemed statistically significant. Clinical heterogeneity was assessed using the Q statistic, tau-square (τ^2^), and I^2^ statistic. Q statistic and τ^2^ interpreted the numerical values, and I^2^ was considered significant at ≥50% [43,44]. To determine the bias in each study, examine its effect on the distribution of summary effect size, and detect outliers, a sensitivity analysis using the “one study removed” method was conducted [45]. In addition, a cumulative meta-analysis was performed to identify the chronological patterns of the effect size of each study [41] (Appendix B).

After determining the effect size of each of the primary studies, their temporal changes were analyzed, and the results before and during the COVID-19 pandemic were compared.

## 3. Results

### 3.1. Study Characteristics

**Figure 1:**
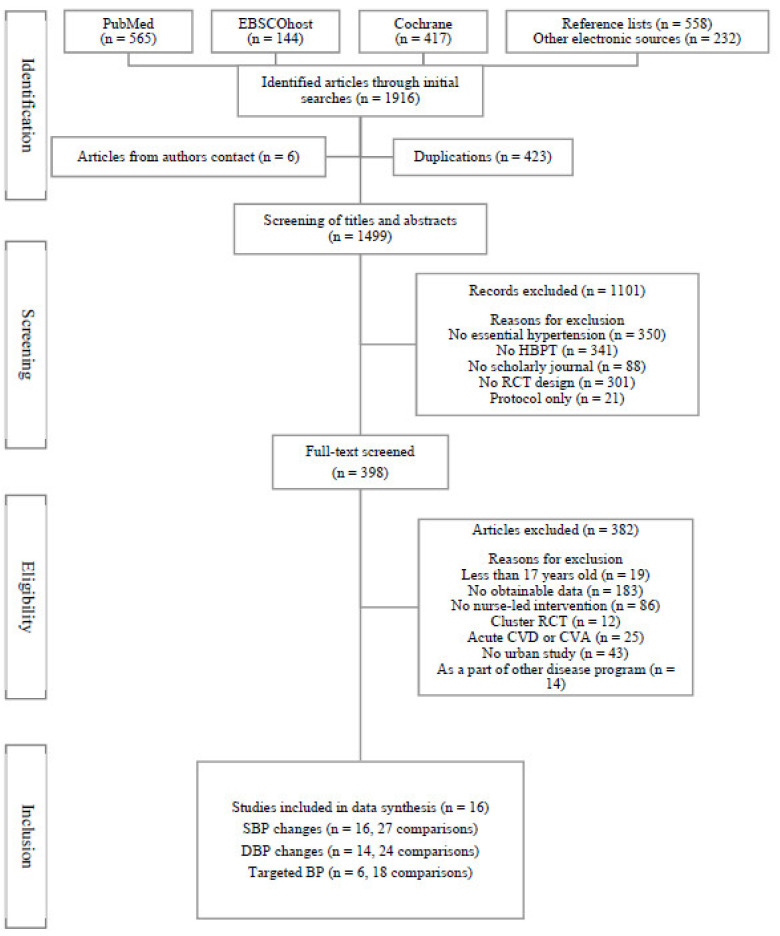
PRISMA flow diagram of the selection of studies included in the systematic review. Note. HBPT, home blood pressure telemonitoring; RCT, randomized controlled trial; CVD, cardiovascular disease; CVA, cerebrovascular accident; SBP, systolic blood pressure; DBP, diastolic blood pressure; BP, blood pressure.

In total, 1916 potentially relevant articles were initially identified from databases, reference lists of each retrieved paper, and other electronic sources (Figure 1). Six datasets were additionally acquired directly from the authors, and 423 duplicates were excluded. The titles and abstracts of 1499 references were screened, and 1101 references were excluded because they were deemed inappropriate for analysis. The full copies of the remaining 398 potentially eligible articles were scrutinized. Of these, 382 studies that did not meet the aforementioned inclusion criteria—that is, studies with participants less than 17 years of age (*n* = 19), no obtainable data (*n* = 183), no nurse-coordinated intervention (*n* = 86), cluster or cross-over RCTs (*n* = 12), patients with life-threatening CVDs or stroke (*n* = 25), studies not performed in an urban setting (*n* = 43), and studies that were part of a research program for another disease (*n* = 14)—were excluded. A total of 27 individual comparisons met the inclusion criteria and were selected as the final material for data synthesis.

In the meta-analysis, the mean length of NC-HBPT was 7.26 months, and the mean age of participants was 61.35 years in the intervention group and 61.62 years in the control group. Of the 27 comparisons, 20 were nurse alone-led cases [21,22,23,26,27,39,40,41,46,47,48,49], and seven cases additionally involved experts from other fields [24,25,27,36,38]. The characteristics of these studies are summarized in Table 1.

The medium of administering the intervention was mobile phones for 2 cases [39], mobile-web for 5 cases [38,40,47], web-based for 5 cases [21,25,37], telephone for 14 cases [22,23,24,26,27,36,46,49], and telephone-linked computers for 1 case [48]. A city was considered metropolitan if its population was at least one million; 11 cases were in metropolitan cities [21,22,23,36,38,39,47,48], and 16 were in smaller cities.

### 3.2. Assessment of Risk of Bias

The selection and performance processes for most trials were appropriate. In the case of attrition or reporting bias domain, there were studies with an unclear or high risk of bias. A primary study at a low risk of bias in at least four domains was deemed to be of high quality [50], and 23 comparisons were identified as having a high risk of bias in fewer than four domains, suggesting that the overall quality of the materials was relatively high.

**Figure 2:**
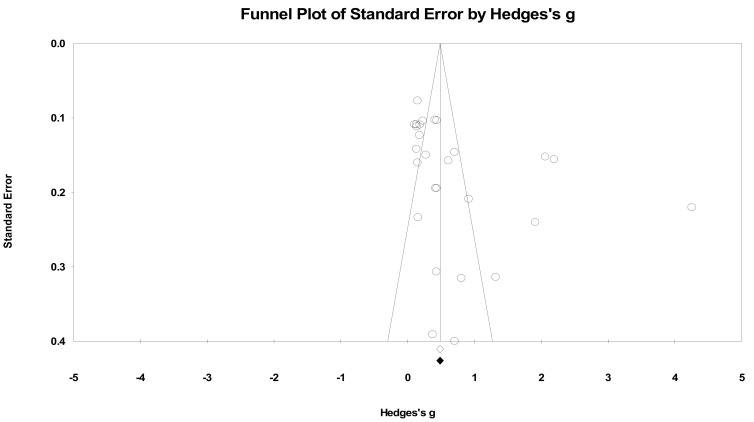
Funnel plot of systolic blood pressure by Hedges’ g (plot observed and imputed), random effects model. Notes. Summary effect size (◇); Summary effect size of imputed studies (◆); Individual studies (○).

**Figure 3:**
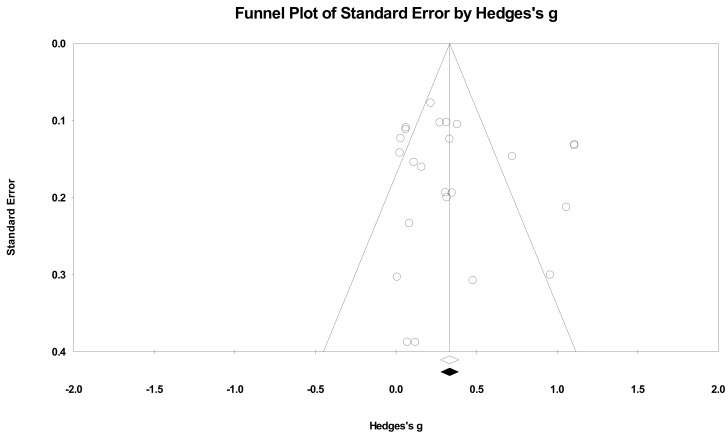
Funnel plot of diastolic blood pressure by Hedges’ g (plot observed and imputed), random effects model. Notes. Summary effect size (◇); Summary effect size of imputed studies (◆); Individual studies (○).

Publication bias was assessed using the trim-and-fill method [51]. The point estimates of SBP based on a funnel plot were as follows: WMD = 5.327 mmHg and *g* = 0.723 (0.445–1.002, *p* < 0.001); there were no trimmed studies (Figure 2). For DBP, *g* = 0.362 (0.222–0.503, *p* < 0.001), which represented a meaningful effect size (Figure 3). The funnel plots of both SBP and DBP showed good visual symmetry, and there were no imputed studies for SBP and DBP. Thus, it was concluded that the potential publication biases did not affect the primary outcomes of the materials included in this study.

**Figure 4:**
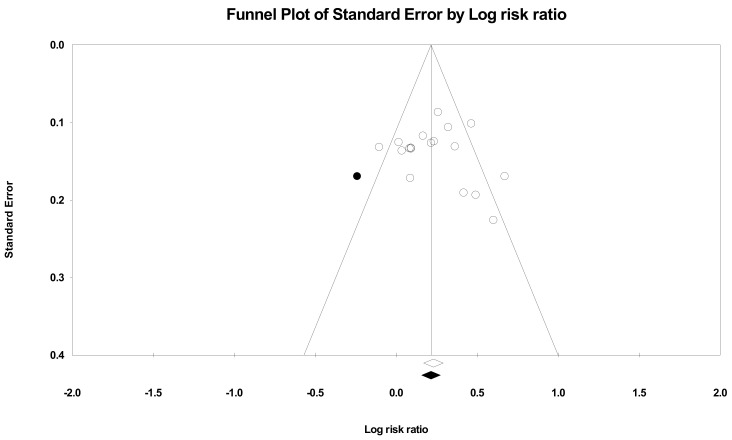
Funnel plot of rate of target blood pressure by log risk ratio (plot observed and imputed), random effects model. Notes. Summary effect size (◇); Imputed study (●); Summary effect size of imputed studies (◆); Individual studies (○).

Publication bias was also assessed for the rate of reaching the target office blood pressure as a secondary outcome. The funnel plot showed good visual symmetry (Figure 4). One study was imputed, but the difference in point estimates was not significant (observed RR, 1.261, vs. adjusted RR, 1.240). Thus, the publication bias by the potentially unpublished study did not affect the RR effect size.

### 3.3. Primary Outcomes

#### 3.3.1. SBP Changes by Nurse-Coordinated HBPT

**Figure 5:**
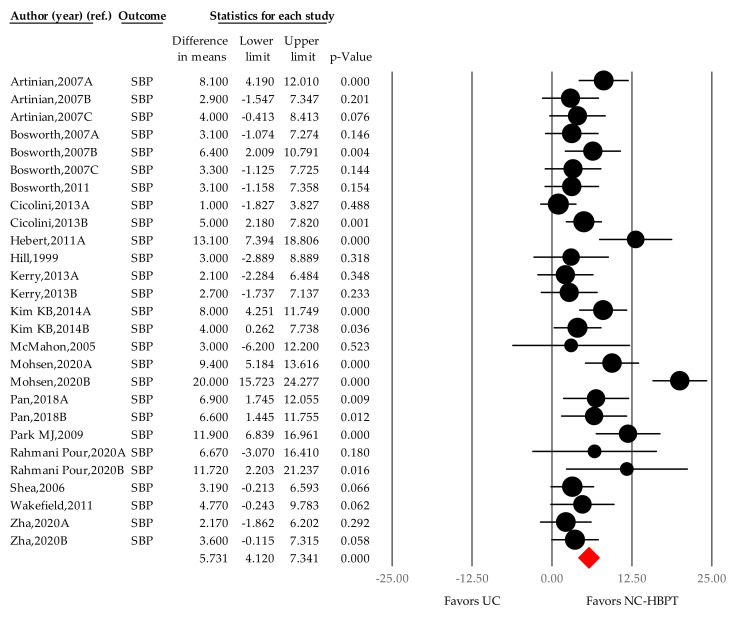
Difference in means of office systolic blood pressure changes by nurse-coordinated intervention. Notes. Point estimate of individual study (*●*); Summary effect size (◆); SBP, systolic blood pressure; UC, usual care; NC-HBPT, nurse-coordinated home blood pressure telemonitoring.

Data were pooled from the 27 comparisons (16 studies) that included 2860 patients in the NC-HBPT group and 2918 patients in the control group, comprising a total of 5778 patients in both groups [21,22,23,24,25,26,27,36,37,38,39,40,46,47,48,49]. A significant reduction in blood pressure was observed in the nurse-led intervention group compared with the UC group (5.731 mmHg; 4.120–7.341; *p* < 0.001; Figure 5), and heterogeneity was significant among the studies (I^2^ = 71.717%; *p* < 0.001). In the sensitivity test [45], the individual studies did not affect the summary point estimates (WMD: 3.822–7.578 mmHg).

**Figure 6:**
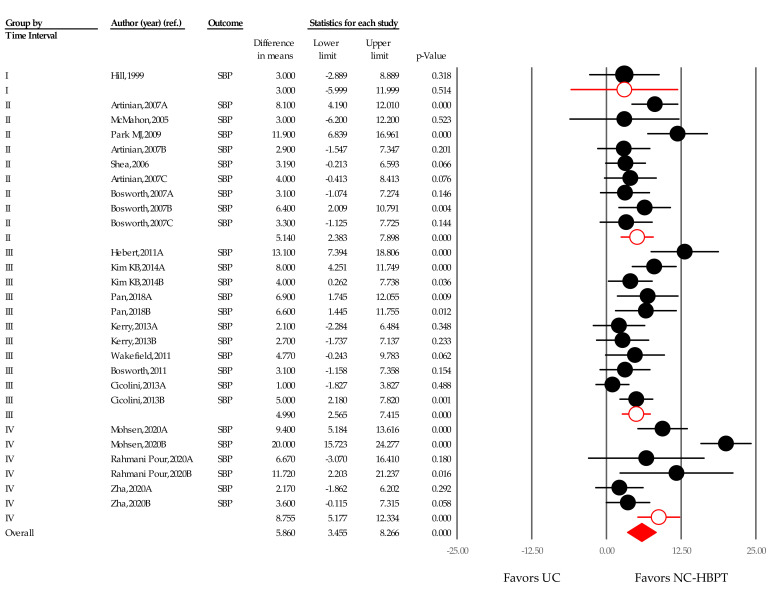
Difference in means of office systolic blood pressure changes over time. Notes. Point estimate of individual study (●); Subgroup (○); Summary effect size (◆); DBP, diastolic blood pressure; UC, usual care; NC-HBPT, nurse-coordinated home blood pressure telemonitoring.

Changes in WMD over time were examined across four time frames. The SBP reduction during time frame I (inception to 2000) was 3.000 mmHg (−5.999 to 11.999; *p* = 0.514) [24]. SBP reductions of 5.150 mmHg (2.383–7.898; *p* < 0.001) and 4.990 mmHg (2.565–7.415; *p* < 0.001) were observed in time frames II (2001–2010) [25,26,27,47,48] and III (2011–2019) [21,23,33,34,35,43,46], respectively (Figure 6). A significant SBP reduction of 8.755 mmHg (5.177–12.334; *p* < 0.001) was observed in time frame IV (2020) [22,39,40], the year in which the COVID-19 pandemic began.

#### 3.3.2. DBP Changes by Nurse-Led Coordination

**Figure 7:**
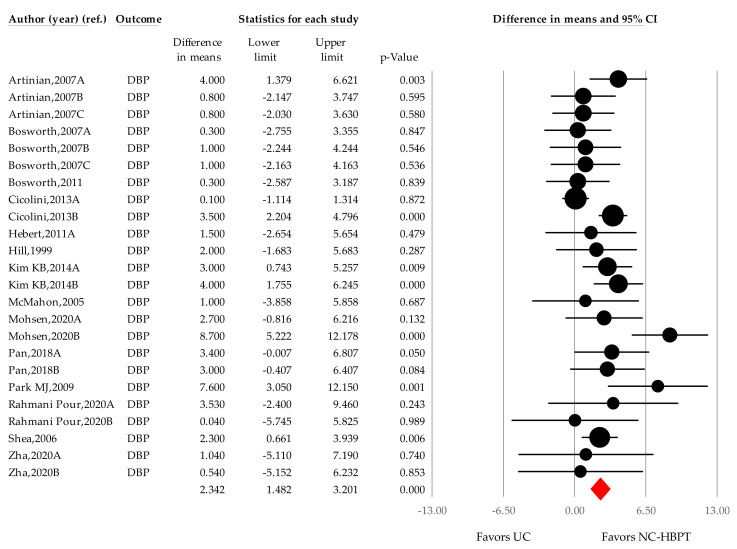
Difference in means of office diastolic blood pressure changes by nurse-coordinated intervention. Notes. Point estimate of individual study (●); Summary effect size (◆); DBP, diastolic blood pressure; UC, usual care; NC-HBPT, nurse-coordinated home blood pressure telemonitoring.

The WMD analysis of DBP was possible using data pooled from 24 comparisons (14 studies) [21,22,23,24,25,26,27,37,38,39,40,46,47,48], and data from 4928 patients (2445 from the NC-HBPT group and 2483 from the UC group) were analyzed. There was a significant decrease in blood pressure in the intervention group compared with the control (2.342 mmHg, 1.482–3.202; *p* < 0.001; Figure 7). Significant heterogeneity was observed between the comparisons (I^2^ = 51.380%; *p* = 0.002). In the sensitivity test, individual studies did not significantly alter the summary effect size (WMD: 1.343–3.359 mmHg).

**Figure 8:**
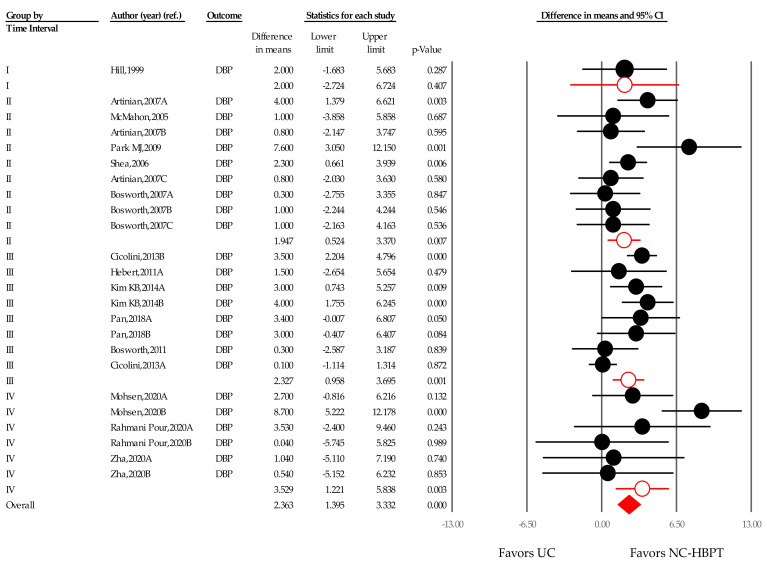
Difference in means of office diastolic blood pressure changes over time. Notes. Summary effect size (◆); Point estimate of individual study (●); Subgroup (○); UC, usual care; NC-HBPT, nurse-coordinated home blood pressure telemonitoring.

The WMD of DBP in the NC-HBPT group was 2.000 mmHg (−2.724 to 6.724; *p* = 0.407) in time frame I [24], 1.947 mmHg (0.524–3.370; *p* = 0.007) in time frame II [25,26,27,47,48], 2.327 mmHg (0.958–3.695; *p* < 0.001) in time frame III [21,23,37,38,46], and 3.529 mmHg (1.221–5.838; *p* = 0.003) in time frame IV [22,39,40], suggesting that the effect of the intervention was statistically greater closer to the COVID-19 outbreak period (Figure 8).

### 3.4. Secondary Outcomes

#### Rate of Reaching the Target Office Blood Pressure

Using 18 available comparisons (6 studies) [21,22,23,27,37,38], we calculated the rate of reaching the target office blood pressure in the NC-HBPT group. Data from 4078 patients (2021 from the intervention group and 2057 from the UC group) showed that the rate of reaching the target office blood pressure was significantly higher in the intervention group than in the UC group (RR 1.261, 1.154–1.378; *p* < 0.001). Heterogeneity between the studies was substantial (I^2^ = 51.6%). In the sensitivity test, study removal did not have a significant effect on the summary effect size (*p* < 0.001; range: 1.133–1.399).

Changes in the rate of reaching the target blood pressure over time were analyzed by combining the data obtained after the year 2000. RR values of 1.101 (1.013–1.198; *p* = 0.024), 1.400 (1.279–1.534; *p* < 0.001), and 1.906 (1.462–2.487; *p* < 0.001) were found for time frames II [27], III [21,23,37,38], and IV [22], respectively, showing a clear improvement rate.

### 3.5. Subgroup Analysis

#### 3.5.1. Size of City

Cities were classified based on their population size, where small- to medium-sized cities had fewer than 1 million residents, and large cities had more than 1 million residents. In small to medium cities (n = 16; participants = 3713) [18,21,24,25,26,27,37,40,49], the decrease in SBP by NC-HBPT was 4.134 mmHg (2.275–5.992, *p* < 0.001; I^2^ = 7.934), while SBP decreased by 8.355 mmHg (5.937–10.773, *p* < 0.001; I^2^ = 82.533) in large cities (n = 11; participants = 2069), compared with the UC group [22,23,36,38,39,47,48] (Appendix C).

#### 3.5.2. Setting

When groups were classified according to the setting in which the study was conducted, the reduction in SBP due to NC-HBPT in primary care clinics (n = 5; participants = 1741) was 3.793 mmHg (0.450–7.136, *p* = 0.026; I^2^ = 0.000) [27,46,48], 4.353 mmHg (1.877–6.829, *p* = 0.001; I^2^ = 34.921) in community health centers (n = 9; participants = 1704) [21,26,38,40], and 7.781 mmHg of SBP (1.375–5.483, *p* < 0.001; I^2^ = 79.627) in hospitals (n = 13; participants = 2337) compared with the UC group [22,23,24,25,36,37,39,47,49].

#### 3.5.3. Duration

For the full duration of NC-HBPT, a total of 5278 people (NC-HBPT group = 2612 vs. UC = 2666) were analyzed. The WMD of SBP was consistently decreased throughout the intervention, by 6.694 mmHg after 3 months (3.644–9.744, *p* < 0.001; I^2^ = 72.511) [21,22,26,38,39,40,47], 6.608 mmHg after 6 months (3.777–9.444, *p* < 0.001; I^2^ = 85.761) [21,22,26,37,38,39,41,50], and 3.573 mmHg at 12 months of intervention (0.796–6.349, *p* = 0.012; I^2^ = 12.877) compared with the UC group [24,25,27,36,37,46,48].

#### 3.5.4. Coordinator’s Profession

A total of 4398 patients were included in the analysis to derive the effectiveness of the nurse-only interventions (n = 20). The decrease in WMD of SBP by nurse alone was 6.132 mmHg (4.262–8.002, *p* < 0.001; I^2^ = 78.074) [21,22,26,27,37,39,40,46,47,48,49]. Cases where nurses collaborated with other professionals (n = 7) included a total of 1384 people and showed a decrease in SBP of 4.465 mmHg (1.122–7.807, *p* = 0.009; I^2^ = 75.373) [24,25,27,36,38]. When classified according to the professions nurses collaborated with, SBP decreased by 2.399 mmHg (−3571–8.369, I^2^ = 0.000) for nutritionists, 3.000 mmHg (−8.682–14.682, I^2^ = 0.000) for lifestyle educators, and 3.000 mmHg (−6.300–12.300, I^2^ = 34.534) for community health workers compared with UC. When the collaboration was with a doctor, SBP was reduced by 6.626 mmHg (1.599–11.652, I^2^ = 67.129) compared with the control group.

#### 3.5.5. Medically Underserved Area

The effect in medically underserved areas reported in each primary study (participants = 5278) was analyzed. In underserved areas (n = 10; participants = 2852), the decrease in SBP was 5.100 mmHg (2.484–7.717, *p* < 0.001; I^2^ = 49.707) [23,24,26,37,40,48], whereas SBP was reduced by 6.150 mmHg (4.041–8.259, *p* < 0.001) in non-marginalized areas (n = 17; participants = 2930) [21,22,25,27,36,38,39,46,47,49]. In the latter areas, the heterogeneity was substantial (I^2^ = 78.192).

## 4. Discussion

This study assessed the effectiveness of NC-HBPT in patients with hypertension, a common NCD, in an urban setting. If remote medical services are defined as delivering patients’ biological information and managing diseases using information and communications technology (ICT) [52], NC-HBPT can be considered a safe, effective, and timely intervention in the current pandemic situation, which has drastically increased the demand for medical resources, in addition to social measures to prevent the spread of COVID-19 [53].

In this meta-analysis, NC-HBPT achieved an SBP reduction of 5.731 mmHg (4.120–7.341, *p* < 0.001) at an average of 7.26 months. A large-scale meta-analysis using individual patient data showed that an SBP reduction of 4 mmHg can reduce the CVD incidence to 10 events/1000 cases in hypertensive patients with a moderate 5-year CVD risk (11–15%) [54]. A previous meta-analysis examining the effects of HBPT in the same setting without consideration of coordinators reported an SBP reduction of 3.482 mmHg (2.459–4.505, *p* < 0.001) [55]. Thus, the increased effect of NC-HPBT in preventing CVD can be considered clinically significant.

When examining the effect of NC-HBPT over time, SBP was relatively low at the beginning of the intervention but increased by 3.000 mmHg (−5.999–11.999, *p* = 0.514), and the maximum reduction of 8.755 mmHg (5.177–12.334, *p* < 0.001) was achieved in 2020, when the pandemic began. Although the number of studies included in time frame I is insufficient and lacks significance, the data cannot be completely ignored, as the aforementioned trend undeniably exists based on the comparison of time frame II values (5.140 mmHg, 2.383–7.898, *p* < 0.001) with those in time frames III and IV. Greater SBP reduction was achieved by NC-HBTP over time. Considering the preference for non-face-to-face contact in 2020 and 2021 due to the COVID-19 pandemic and the increased demand for remote medical services, it can be extrapolated from our results that an increase in the potential effectiveness of remote medical services with nurse coordination is possible.

Based on evidence from previous literature suggesting that NC-HBPT is effective [3,20], we examined the effect of a nurse-alone intervention through 20 comparisons [21,22,23,26,27,37,39,40,46,47,48,49]. HBPT coordinated by a nurse alone achieved an SBP reduction of 6.132 mmHg (4.262–8.002, *p* < 0.001), which was not inferior to the mean reduction of interventions by all coordinators (5.731 mmHg). Moreover, compared with NC-HBPT additionally coordinated by an expert from another field, HBPT was even more effective when a physician was involved in the intervention (n = 3; 6.626 mmHg, 1.599–11.652) [27,38]. HBPT showed limited effectiveness when coordinated by community health workers (3.000 mmHg, −6.300–12.300) [24], nutritionists (2.399 mmHg, −3.571–8.369) [36], or lifestyle educators (3.000 mmHg, −8.682–14.682) [25].

We also found that NC-HBPT was associated with a larger reduction in DBP (2.342 mmHg, 1.482–3.201, *p* < 0.001) than HBPT in the same setting but without consideration of coordinators (1.638 mmHg, 1.084–2.192, *p* < 0.001) [55]. Similar patterns were observed in DBP and SBP changes over time. A higher reduction in DBP of 3.529 mmHg (1.221–5.838, *p* = 0.003) was observed in time frame IV [22,39,40] than in time frame II (1.947 mmHg, 0.524–3.370; *p* = 0.007) [25,26,27,47,48].

HBPT coordinated by a nurse alone achieved a DBP reduction of 2.389 mmHg (1.393–3.384, *p* < 0.001) [21,22,23,24,26,27,37,39,40,47,48,49], and additional coordination by a doctor achieved a DBP reduction of 2.440 mmHg (−0.166–5.047, *p* = 0.066) [26,38], showing a significantly greater DBP reduction when HBPT is additionally coordinated by experts from other specific fields than in NC-HBPT [24,25,26,38]. Although the reason for the differences in blood pressure reductions according to the profession of the additional coordinator is unclear, it may have to do with social and organizational factors, the coordinator’s level of medical knowledge and experience, and similarity in the education received by the coordinators [56,57]. However, since the number of cases in which NC-HBPT was coordinated by experts from other fields was small and the results were not statistically significant, further research is needed to more accurately determine the validity of the effect of the intervention.

Palmas et al. (2006) reported that a lack of awareness of the benefits of remote medical technologies and the burden of using these technologies have contributed to the low expectations for remote medical services in urban areas [58]. However, the high percentage of hypertensive patients who are highly susceptible to COVID-19 and the environmental factors that are found in densely populated cities contribute to excessive medical demands that cannot be handled by traditional medical systems [59]. Therefore, the importance of remote monitoring technology as a means to efficiently provide medical services with limited resources is being increasingly emphasized. To the best of our knowledge, this is the first meta-analysis to examine the effect of NC-HBPT on urban hypertensive patients over time. We have derived meaningful results regarding the benefits of nurse-coordinated interventions.

In this study, the heterogeneity of the summary effect sizes was found to be substantial. Specifically, the I^2^ for SBP and DBP were 71.717% (*p* < 0.001) and 51.380% (*p* = 0.002), respectively. Thus, the authors applied a random effects model to the analysis, which did not completely remove the heterogeneity. Therefore, subgroup analysis was performed to assess the causes of the heterogeneity, and several moderators were explored that revealed clinical implications, along with evaluation of heterogeneity. 

Despite selecting well-founded blinded RCTs through a transparent and systematic process and deriving solid and integrated results for the primary outcomes without publication biases, our study has some limitations. First, an extensive literature search was conducted on reliable and relevant databases using a structured formula, but the number of studies included was low. By building a more precise search formula, the reliability of our results could be improved. Second, since each time frame did not contain an equal number of comparisons, the analysis results for different periods were not based on the same number of studies. Including as many studies as possible, with an equal number of studies per time frame, can overcome this limitation. Third, while the researchers reasonably set the duration of each time frame to 9 and 10 years according to the technological changes and historical events to explore temporal patterns of outcomes of NC-HBPT, the distinction between each time frame may not have been clear. Thus, it may be necessary to set the time frames based on the turning points of ICT development to increase the precision of the findings. Lastly, for studies that compared interventions with different coordinators or lengths of follow-up, each comparison was counted as an independent primary study, but there were cases where multiple comparisons were included in one study. Although there was substantial heterogeneity between each comparison, and no statistical errors were observed, there may be a lack of optimal scientific robustness due to the methodological limitations of a random effects model. This limitation can also be overcome by updating the results based on a larger number of studies.

## 5. Conclusions

This study revealed that NC-HBPT for urban hypertensive patients can deliver clinically and statistically good BP reduction in terms of avoiding CVD outbreak when compared with UC. Our findings may have meaningful implications for policymakers in urban areas that are planning to introduce remote monitoring systems or in areas with inefficient telemedicine systems. However, some included studies in this analysis lack quality. Thus, although the evidence for the benefit of NC-HBPT was found in this review, further research is necessary on the nurses’ roles as coordinators. Additionally, future work must consider the effect of multiple variables on NC-HBPT for more efficient implementation of the intervention system in urban areas.

## Figures and Tables

**Table 1:** Characteristics of included individual studies.

Study	Number of Subjects (UC vs. HBPT Group)	Inclusion Criteria	The Profession of Coordinator	Name of City (Country)	Size of Urban Population (Over 1,000,000)	Description of Intervention Pathway	Additional Support	Main Outcome Measures
Artinian 2007A [26]	193/194	African-American hypertensive patients	Trained registered nurse	Detroit (USA)	No (916,952 in 2007)	Telephonic transmission	Telecounseling call and patient education	Changes in office BP
Artinian 2007B [26]	157/164	African-American hypertensive patients	Trained registered nurse	Detroit (USA)	No (916,952 in 2007)	Telephonic transmission	Telecounseling call and patient education	Changes in office BP
Artinian 2007C [26]	169/167	African-American hypertensive patients	Trained registered nurse	Detroit (USA)	No (916,952 in 2007)	Telephonic transmission	Telecounseling call and patient education	Office BP changes
Bosworth 2007A [27]	150/150	Hypertensive patients who are using a BP-lowering medication with poor (inadequate) BP control	Intervention nurses	Durham (USA)	No (217,847 in 2007)	Home BP monitoring + Behavioral intervention	Tailored behavioral intervention	Improved rates of BP control
Bosworth 2007B [27]	150/150	Hypertensive patients who are using a BP-lowering medication with poor (inadequate) BP control	Intervention nurses supervised with a physician	Durham (USA)	No (217,847 in 2007)	Home BP monitoring + Medication management	Medication management with a validated decision support system (DSS)	Improved rates of BP control
Bosworth 2007C [27]	150/150	Hypertensive patients who are using a BP-lowering medication with poor (inadequate) BP control	Intervention nurses	Durham (USA)	No (217,847 in 2007)	Home BP monitoring + Combined intervention	Combined behavioral and medication management	Improved rates of BP control
Bosworth 2011 [46]	137/127	Treated hypertensive patients	Registered nurse	Durham (USA)	No (228,354 in 2010)	Telephonic transmission	Behavioral management	Change in BP control, SBP, and DBP
Cicolini 2013A [21]	98/100	Treated and untreated hypertensive patients	Registered nurse	Chieti (Italy)	No (51,484 in 2011)	Web-based	Email reminder and phone call	1. Changes in BP2. BMI, alcohol consumption, cigarette smoking, adherence to therapy
Cicolini 2013B [21]	98/100	Treated and untreated hypertensive patients	Registered nurse	Chieti (Italy)	No (51,484 in 2011)	Web-based	Email reminder and phone call	1. Changes in BP2. BMI, alcohol consumption, cigarette smoking, adherence to therapy
Hebert 2011A [23]	83/85	Uncontrolled hypertensive patients	Registered nurse	New York (USA)	Yes (8,174,959 in 2010)	Telephonic transmission	Information on the use of home BP monitor	BP reduction
Hill 1999 [24]	77/78	Black or African-American hypertensive young male resident within the Johns-Hopkins Hospital catchment area	Nurse-community health worker team (registered nurse and health worker team)	Baltimore (USA)	No (763.014 in 1990)	Telephonic transmission	Individualized counseling, monthly telephone call, and a home visit (educational-behavioral intervention)	Changes in office BP
Kerry 2012A [36]	169/168	Hypertensive patients with history of stroke or transient ischemic attack	Nurse	London (UK)	Yes (8,135,667 in 2011)	Telephonic transmission	Nurse-led telephone support	Reduction of SBP
Kerry 2012B [36]	169/168	Hypertensive patients with history of stroke or transient ischemic attack	Nurse	London (UK)	Yes (8,135,667 in 2011)	Telephonic transmission	Nurse-led telephone support	Reduction of SBP
Kim KB 2014A [37]	192/191	Uncontrolled Korean-American hypertensive seniors	Bilingual RNs and nutritionist	Ellicott City (USA)	No (68,507 in 2014)	Web-based	2 h weekly education and training for 6 weeks and monthly telephone counseling	Changes in SBP and DBP
Kim KB 2014B [37]	187/185	Uncontrolled Korean-American hypertensive seniors	Bilingual RNs and nutritionist	Ellicott City (USA)	No (68,507 in 2014)	Web-based	2 h weekly education and training for 6 weeks and monthly telephone counseling	Changes in SBP and DBP
McMahon 2005 [25]	35/37	Poorly controlled diabetics and hypertensive patients	Advanced practice nurse and certified diabetes educator	Boston (USA)	No (559,034 in 2005)	Web-based	Telephone to encourage website usage	Changes in HbA_1c_, BP, lipid profiles
Mohsen 2019A [22]	50/50	Hypertensive patients who are on antihypertensive medication	Staff nurse	Shibin El Kom (Egypt)	No (249,611 in 2018)	Telenursing	Follow-up phone calls	Reducing arterial blood pressure and patients’ anthropometric measurement
Mohsen 2019B [22]	50/50	Hypertensive patients who are on antihypertensive medication	Staff nurse	Shibin El Kom (Egypt)	No (249,611 in 2018)	Telenursing	Follow-up phone calls	Reducing arterial blood pressure and patients’ anthropometric measurement
Pan 2018A [38]	55/52	Hypertensive patients with uncontrolled BP	GP, a hypertensionspecialist, a general nurse, an information manager	Beijing (China)	Yes (19,612,368 in 2010)	Smartphone application	Follow-up phone calls	The reduction in systolic blood pressure
Pan 2018B [38]	55/52	Hypertensive patients with uncontrolled BP	GP, a hypertensionspecialist, a general nurse, an information manager	Beijing (China)	Yes (19,612,368 in 2010)	Smartphone application	Follow-up phone calls	The reduction in systolic blood pressure
Park MJ 2009 [47]	21/28	Obese hypertensive patients	Registered nurse	Seoul (S. Korea)	Yes (10,208,302 in 2009)	Mobile or internet transmission	Short message service by cellular phone and internet recommendation	Changes in BP, body weight, waist circumference, and serum lipid profile
Rahmani Pour 2019A [39]	21/21	Hypertensive patients, use of antihypertensive medications	Instructors of a faculty of nursing and midwifery and several cardiologists	Tehran (Iran)	Yes (8,693,706 in 2016)	Short message service	Communicate with the first author	Improved treatment adherence, no significant differences among the groups with respect to the baseline SBP and DBP
Rahmani Pour 2019A [39]	21/21	Hypertensive patients, use of antihypertensive medications	Instructors of a faculty of nursing and midwifery and several cardiologists	Tehran (Iran)	Yes (8,693,706 in 2016)	Non-ISMS	Follow-up	No significant differences among the groups with respect to the baseline SBP and DBP
Shea 2006 [48]	347/333	Diabetic hypertensive patients	Nurse case manager	New York (USA)	Yes (8,143,197 in 2005)	Telephone-linked web-enabled computer system	Web-based messaging	Changes in HbA_1c_, BP, and cholesterol level
Wakefield 2011 [49]	97/83	Type 2 diabetics and hypertensive patients	Registered nurse	Iowa City (USA)	No (68,036 in 2010)	Telephonic transmission	Telephonic transmission and nurse care management	Changes in HbA_1c_ and SBP
Zha 2020A [40]	13/12	Underserved and vulnerable urban population on HTN medication with diagnosed uncontrolled hypertension	Two nurses in the community health center	Newark (USA)	No (281,764 in 2016)	Mobile Health (Smart phone or tablet)	Three training sessions	No significant change in systolic BP, the potential to facilitate hypertension management
Zha 2020B [40]	13/12	Underserved and vulnerable urban population on HTN medication with diagnosed uncontrolled hypertension	Two nurses in the community health center	Newark (USA)	No (281,764 in 2016)	Mobile Health (Smart phone or tablet)	Three training sessions	No significant change in systolic BP, the potential to facilitate hypertension management

Notes. UC, usual care; HBPT, home blood pressure telemonitoring; GP, general practitioner; BP, blood pressure; SBP, systolic blood pressure; DBP, diastolic blood pressure; HTN, hypertension; Non-ISMS, non-interactive short message service; BMI, body mass index; HbA1c, glycated hemoglobin.

**Figure A1:**
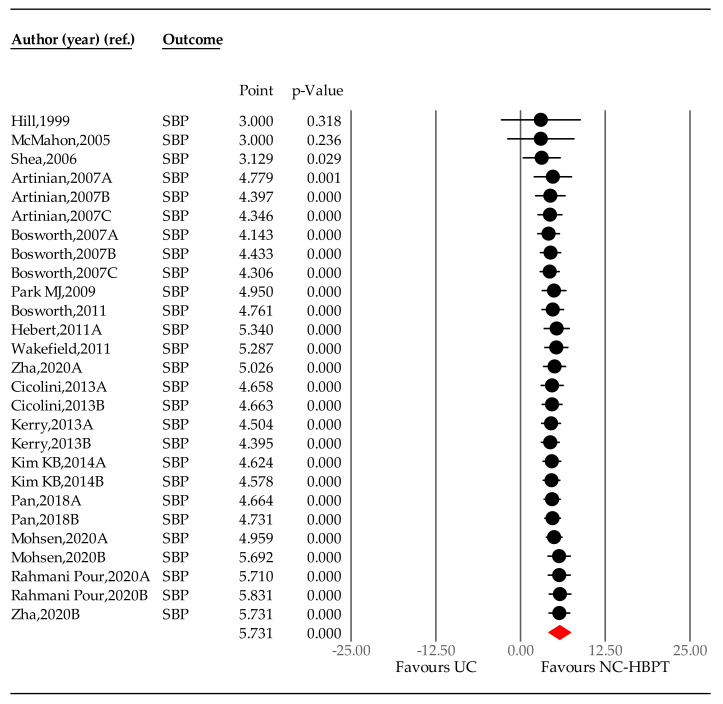
Cumulative meta-analysis presenting the summative effect at each time point. Note. SBP, systolic blood pressure; UC, usual care; NC-HBPT, nurse-coordinated home blood pressure telemonitoring; Summary effect size (◆); Point estimate of individual study (●).

## Appendix A. Searching Formula

(((((((“Hypertension”[Mesh]) OR ((hypertensi*) OR high blood pressure))) AND ((((((“Urban Population”[Mesh]) OR “Urban Health”[Mesh]) OR “Urban Health Services”[Mesh]) OR “Cities”[Mesh])) OR ((((urban*) OR city) OR cities) OR central cit*)))) AND (((((“Telemedicine”[Mesh]) OR “Telemetry”[Mesh]) OR “Blood Pressure Monitoring, Ambulatory”[Mesh])) OR (((((((((((telemedicine) OR telemetry) OR telenurs*) OR telemonitor*) OR eHealth) OR telehealth) OR remote monitor*) OR technology) OR telephone) OR smartphone) OR internet)))) AND (((((((((randomised controlled trial) OR randomized controlled) OR controlled clinical trial)) OR ((((((((randomised[Title/Abstract]) OR randomized[Title/Abstract]) OR placebo[Title/Abstract]) OR drug therapy[Title/Abstract]) OR groups[Title/Abstract]) OR clinical trials as topic[Title/Abstract]) OR randomly[Title/Abstract]) OR trial[Title/Abstract]))) NOT cluster randomized controlled trials)) NOT cross over study)

## Appendix C. Subgroup Analysis

**Table A1:** Subgroup analysis.

Category	Number of Studies	Summary WMD of SBP, mmHg (95% CI)	Heterogeneity, I^2^ (%) by FEM	*p*-Value of I^2^
Overall	27	5731	71.717	*p* < 0.001
City size (population)				
Under 1 million	16	4.134 (2.275–5.992)	7.934	*p* = 0.063
Over 1 million	11	8.355 (5.937–10.773)	82.533	*p* < 0.001
Setting				
Primary care clinic	5	3.793 (0.450–7.136)	0.000	*p* = 0.086
Community health center	9	4.353 (1.877–6.829)	34.921	*p* = 0.039
Hospital	13	7.781 (5.483–10.079)	79.627	*p* < 0.001
Duration (month)				
3 or less	8	6.694 (3.644–9.744)	72.511	*p* = 0.001
6	8	6.608 (3.771–9.444)	85.761	*p* < 0.001
12	9	3.573 (0.796–6.349)	12.877	P = 0.042
Medically underserved area				
Not underserved	17	6.150 (4.041–8.259)	78.192	*p* < 0.001
Underserved	10	4.424 (2.484–7.717)	49.707	*p* = 0.036
Collaboration with other professionals				
No (Nurse-alone)	20	6.132	78.074	*p* < 0.001
Yes	7	4.465	75.373	*p* = 0.633

Note. WMD, weighted mean difference; SBP, systolic blood pressure; CI, confidence interval; FEM, fixed effect model.
